# Attraction Effects in Honorific Agreement in Korean

**DOI:** 10.3389/fpsyg.2016.01302

**Published:** 2016-08-31

**Authors:** Nayoung Kwon, Patrick Sturt

**Affiliations:** ^1^Department of English Language and Literature, Konkuk UniversitySeoul, South Korea; ^2^Psychology, School of Philosophy, Psychology and Language Sciences, University of EdinburghEdinburgh, UK

**Keywords:** retrieval, attraction effects, subject-verb honorifics agreement, Korean, intrusion effects, eye-tracking

## Abstract

Previous studies have suggested that sentence processing is mediated by content-addressable direct retrieval processes (McElree, 2000; McElree et al., 2003). However, the memory retrieval processes may differ as a function of the type of dependency. For example, while many studies have reported facilitatory intrusion effects associated with a structurally illicit antecedent during the processing of subject-verb number or person agreement and negative polarity items (Pearlmutter et al., 1999; Xiang et al., 2009; Dillon et al., 2013), studies investigating reflexives have not found consistent evidence of intrusion effects (Parker et al., 2015; Sturt and Kwon, 2015; cf. Nicol and Swinney, 1989; Sturt, 2003). Similarly, the memory retrieval processes could be also sensitive to cross-linguistic differences (cf. Lago et al., 2015). We report one self-paced reading experiment and one eye-tracking experiment that examine the processing of subject-verb honorific agreement, a dependency that is different from those that have been studied to date, in Korean, a typologically different language from those previously studied. The overall results suggest that the retrieval processes underlying the processing of subject-verb honorific agreement in Korean are susceptible to facilitatory intrusion effects from a structurally illicit but feature-matching subject, with a pattern that is similar to subject-verb agreement in English. In addition, the attraction effect was not limited to the ungrammatical sentences but was also found in grammatical sentences. The clear attraction effect in the grammatical sentences suggest that the attraction effect does not solely arise as the result of an error-driven process (cf. Wagers et al., 2009), but is likely also to result from general mechanisms of retrieval processes of activating of potential items in memory (Vasishth et al., 2008).

## Introduction

Previous studies have suggested that dependency resolution during on-line sentence processing is mediated by a content-addressable retrieval process (McElree et al., 2003; Lewis and Vasishth, 2005; Lewis et al., 2006; Van Dyke and McElree, 2006). These studies, however, have only examined Indo-European languages. Thus, the current study examines the processing of a dependency in Korean, a typologically different language from those previously studied to investigate cross-linguistic generality of retrieval processes. In particular, we examine the processing of subject-verb honorific agreement.

### Retrieval process underlying on-line sentence processing

Retrieval processes have been broadly characterized in terms of two mechanisms: Search (e.g., Sternberg, 1966) and content-addressable direct access (e.g., Kintsch, 1970). The defining feature of the search model is that items are individually stored in memory and when prompted, each item is retrieved and evaluated until a target item is retrieved. Accordingly, with a search model, the number of items in memory or the number of intervening materials between the target item and a test probe are predicted to affect response latencies. For example, for an accurate “no” response to be made in a recognition task, an exhaustive search should be completed comparing each item in memory with a probe before the response, and thus slow responses are predicted. On the other hand, according to the content-addressable direct retrieval, the access to the target item is cued by a test probe, and this allows direct access to the target item in memory. Thus, with the direct access model, the number of items in memory or the number of intervening items is not predicted to affect retrieval speed although it might affect accuracy as the representation of an item in memory could be degraded with more items to encode and with more time for the representation to decay.

Among these two models, recent studies found evidence in support of the content-addressable direct access mechanisms for on-line sentence processing (McElree, 2000; McElree et al., 2003). For example, McElree (2000) examined the processing of sentences with various numbers of relative clauses (e.g., 1 relative clause: *This was the book that the editor admired/amused vs*. 2 relative clauses: *This was the book that the editor* *who the receptionist married* *admired/amused vs*. 3 relative clauses: *This was the book that the editor* *who the receptionist who quit married* *admired/amused*), using a speed-accuracy tradeoff procedure (SAT henceforth). During this procedure, participants were asked to evaluate acceptability of the sentences at designated times between 50 and 3000 ms after the onset of the sentence-final verb, *admired or amused*, at which the replaced filler, *the book*, was supposedly retrieved. The results showed that the number of interpolated items (i.e., the numbers of embedded relative clauses) did not affect the speed of performance, suggesting that the target item (e.g., *the book*) was equally quickly accessed for these sentences. On the other hand, accuracy declined with more interpolated items. These results were taken to support the content-addressable direct retrieval model.

Further support for the content-addressable direct retrieval hypothesis comes from the so-called *attraction* effect. Pearlmutter et al. (1999) examined the processing of subject-verb agreement in English as in (1), and found that processing difficulty was not just affected by the linguistic items participating in a dependency (i.e., subject and verb: *The key* and *was/were*) but also by a distractor item that is not relevant for the subject-verb agreement (i.e., the complement NP to the preposition: *The cabinet*).

(1) Pearlmutter et al. (1999).
a. The key to the cabinet was rusty from many years of disuse.
b. The key to the cabinets was rusty from many years of disuse.
c. ^*^The key to the cabinet were rusty from many years of disuse.
d. ^*^The key to the cabinets were rusty from many years of disuse.

That is, while there was a main effect of grammaticality, with ungrammatical sentences taking longer to read (1-c,d) than their grammatical counterparts (1-a,b), the effect was accompanied by an interaction with the type of distractor NP; the processing difficulty due to ungrammaticality was greatly reduced when the distractor NP matched the verb in the number features (1-d: *The cabinets -were*) compared with when it did not (1-c: *The cabinet -were*). Under the content-addressable direct retrieval hypothesis, the attraction effect is accounted for in terms of mis-retrieval of a distractor during retrieval processes. That is, as retrieval cues (e.g., plural form of a verb, *were*) activates any linguistic item in memory with matching features even when the feature match is partial, a distractor with matching feature could be mis-retrieved, leading to “grammatical illusions” and reducing processing difficulty. In the literature, the facilitatory effects of a distractor is also known as *facilitatory intrusion*, or *facilitatory interference* (for related work in production, see Bock and Miller, 1991; Bock and Cutting, 1992; Bock and Eberhard, 1993; Vigliocco and Nicol, 1998; Hartsuiker et al., 2001; Haskell and MacDonald, 2003; Thornton and MacDonald, 2003; for related works on similarity-based interference, see Lewis, 1996; Gordon et al., 2001, 2004, 2006).

The attraction effect in subject-verb number agreement has been further replicated in several other studies (Nicol et al., 1997; Thornton and MacDonald, 2003; Dillon et al., 2013; Tanner et al., 2014; Lago et al., 2015; Parker et al., 2015) even in cases where the distractor does not linearly intervene between the subject and verb (Wagers et al., 2009). In addition, the attraction effect has been found in studies of the processing of negative polarity items (NPI; Drenhaus et al., 2005; Vasishth et al., 2008; Xiang et al., 2009). Although the NPI “ever” should be licensed by a c-commanding negative licensor in German, as in (2-a), its processing profile has also been found to be affected by an inaccessible (non-c-commanding) negative licensor (Drenhaus et al., 2005; Vasishth et al., 2008).

(2) English translation of German experimental sentences in Drenhaus et al. (2005).
a. Accessible NPI licensor & NPI:**No pirate** [who a roast eaten had] was **ever** thrifty.
b. Inaccessible NPI licensor & NPI:^*^A pirate [who **no roast** eaten had] was **ever** thrifty.
c. No NPI licensor:^*^A pirate [who a roast eaten had] was ever thrifty.

Thus, while processing penalty was found for the two unacceptable conditions without an accessible NPI licensor (2-b,-c) compared with the acceptable condition (2-a), the processing difficulty was greatly reduced in (2-b) compared with (2-c), due to the presence of a structurally illicit negative licensor in (2-b). In (2-c), there is no such potential licensor available, resulting in a greater processing penalty. These results are compatible with the proposal that sentence processing is mediated by content-addressable direct retrieval processes, during which syntactic and/or semantic features are used in parallel as retrieval cues (McElree, 2000; McElree et al., 2003).

However, studies investigating reflexives have not found consistent evidence of attraction, compared with studies investigating subject-verb agreement, or negative polarity items. Thus, while Sturt and Kwon (2015) found facilitatory intrusion effects of a distractor in sentences with nominal control (e.g., *John's agreement with Amy to be kind to himself…*) and raising (e.g., *John seemed to Amy to be kind to himself…*), the effects were only found in later parsing stages of reflexives (e.g., control: In the first-pass and go-past times at a spillover region; raising: In the second pass times at the critical reflexive position). In addition, the same study did not find comparable facilitatory effects of a distractor in coordinate sentences (e.g., *John did not trust Amy but was kind to himself …*). Likewise, Parker et al. (2015) reported facilitatory intrusion effects of a distractor when they manipulated animacy feature of a potential antecedent for a reflexive (e.g., The doctor/discovery that the researcher/report liked …himself…), but no attraction effect was found when they manipulated a relatively weak semantic feature (i.e., gender rather than animacy; The harpist/drummer that the diva/guitarist liked …herself…). In fact, a number of studies have failed to find the attraction effect in the processing of reflexives (Nicol and Swinney, 1989; Xiang et al., 2009; Clackson et al., 2011; Dillon et al., 2013; for similar findings with bound variable pronouns, see Kush et al., 2015). This has led several authors to assume that the processing of reflexives is relatively “immune” to the attraction effect (for a related discussion, see Dillon et al., 2013; Parker et al., 2015).

These differences in different types of dependencies (e.g., number or person agreement and NPI vs. anaphora), however, were not predicted given the superficial surface similarities between them, raising a possibility that retrieval processes are sensitive to different types of a cue (e.g., animacy vs. gender), types of a dependency (e.g., number or person agreement, NPI vs. anaphor), and types of a construction being processed (e.g., raising or control vs. coordination) and potentially also sensitive to cross-linguistic differences (cf. Lago et al., 2015). This calls for further investigations of retrieval processes using a dependency of still different nature in a typologically different language from those previously studied, and this is one of the motivations for the present study on honorific agreement in Korean. As discussed in detail in the next section, the honorific dependency in Korean is not obligatory unlike grammaticalized subject-verb agreement in English. Instead, it is based on pragmatic features signaled by pragmatic or world knowledge. Accordingly, honorific agreement in Korean provides a good testing ground to evaluate the generality of retrieval processes.

The second goal of the study concerns the nature of attraction effects: Whether attraction effects result from general working memory principles or from error-driven processing mechanisms. According to the content-addressable direct access hypothesis, it is assumed that retrieval cues will activate any linguistic item in memory with matching features, even where the feature match is partial. The levels of activation for these items could differ depending on the weights associated with each cue, but nonetheless the activated items can potentially affect the dependency resolution to some degree, given that the activation level exceeds the retrieval threshold (Vasishth et al., 2008). This would mean that attraction affects the initial stages of dependency formation, and could potentially apply to both grammatical and ungrammatical dependencies. The second possibility is also compatible with the content-addressable direct retrieval process but makes more specific predictions. That is, if the attraction effect is an error-driven processing mechanism, the effect would be found only for ungrammatical sentences, and only at a relatively late stage of processing, following the initial reaction of the parser to ungrammaticality. In fact, in a self-paced reading study, Lago et al. examined grammaticality and attraction effects for the entire distributions of reaction times at a spill-over word and found that grammaticality effects were generally observed earlier than attraction effects. In addition, attraction effects have been typically found in ungrammatical sentences but not in grammatical sentences (Pearlmutter et al., 1999; Wagers et al., 2009; Dillon et al., 2013; Lago et al., 2015; cf. Van Dyke, 2007). These results are compatible with the hypothesis that the attraction effects arise as the result of error-driven processing when participants have already experienced difficulty due to feature mismatches during the processing of a dependency (Wagers et al., 2009; Lago et al., 2015). However, many previous studies have been conducted using self-paced reading time, which has a relatively low temporal resolution. Thus, further investigation is needed to examine this question, using eye-tracking, a method that allows higher temporal resolution. This is another question that we aim to examine in this study.

In summary, dependency resolution during on-line sentence processing is mediated by a content-addressable retrieval process. This is supported many studies showing facilitatory intrusion effects of a structurally illicit antecedent during the processing of subject-verb number or person agreement, NPIs, and reflexives in English. However, the intrusion effects have been shown to be sensitive to different types of cues and dependencies, and could possibly also be sensitive to typological differences. In this study, we investigate the processing of subject-verb honorific agreement in Korean to further investigate generality of the process (Experiments 1, 2). While doing so, we also aim to investigate the detailed time-course of the attraction effect in relation to the grammaticality effect, using an eye-tracking method (Experiment 2).

### Relevant grammatical sketches of Korean

Unlike the Indo-European languages that have been examined in most previous studies of attraction, Korean does not have a rich agreement system, and morphological cues on verbs do not serve as strong cues for identifying subjects, since the verb in Korean does not agree with its subject in person or number (Huang, 1984; Kwon and Sturt, 2013)^1^. An exception to this is the honorific marker, -*si*-. It attaches to a verb and has to agree with the subject in honorific feature (e.g., *grandpa*) and cannot be used with a subject of low social status (e.g., *kid*). Importantly, however, it is not grammatically motivated and thus the omission of the marker does not lead to ungrammaticality of the sentence. Instead, honorifics only provide information on the perceived relative social hierarchical status of the subject in relation to the speaker. Thus, although the honorific system in Korean involves a kind of subject-verb agreement, the nature of this agreement is different from that of verb-subject agreement in English. The honorific dependency in Korean is based on pragmatic features, and is not obligatory, while subject-verb agreement in English is obligatory, and based on grammaticalized number and person features.

Korean has a productive case system, such that grammatical particles are suffixed to NPs to encode grammatical and semantic relationships between NPs. For example, subjects are typically marked with nominative case particle -*i/ka*, objects with accusative case particles -*ul/lul* and NPs with a topic reading are marked with topic marker -*un/nun*.^2^ In addition, there is a deferential nominative particle, -*kkeyse*, which can be used to mark subject NPs with honorific features. Like the verbal honorific marker -*si*-, the deferential nominative particle -*kkeyse* is also optionally used. Thus, subjects with high social status can be marked with -*i/ka* as well as -*kkeyse* and so the choice of a nominative particle could signal perceived social status of the sentential subject relative to that of a speaker.

In the experiments reported below, we report a self-paced reading experiment (Experiment 1) and an eye-tracking experiment (Experiment 2) that investigate attraction effects in Korean subject-verb honorific dependencies.

## Experiment 1

Experiment 1 used a self-paced reading time method to investigate the retrieval processes underlying the processing of subject-verb honorific agreement in Korean.

### Participants

Thirty seven native speakers of Korean (mean age 21 years) participated in the study. All had normal or corrected-to-normal vison. At the time of the participation, they were undergraduate students enrolled in Konkuk University, Korea. They received W10000 per hour for their participation.

### Materials

**Figure 1:**
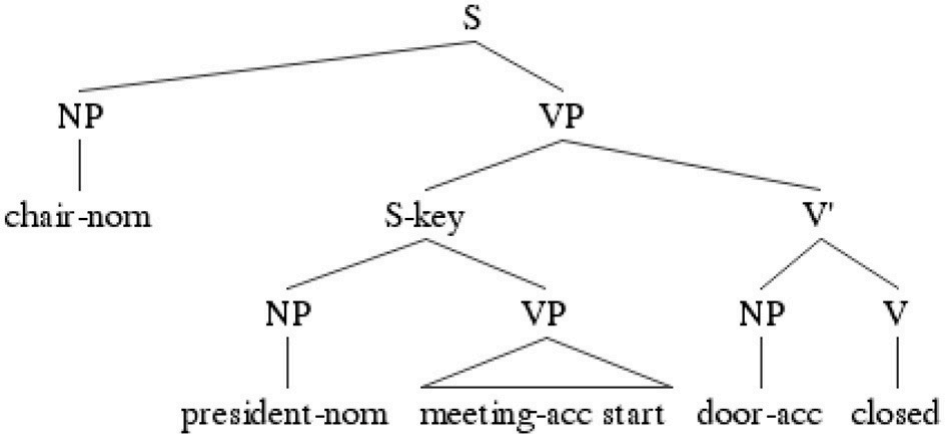
**Tree structure of the target construction**.

The experimental sentences were composed of a main clause and a subordinate adjunct clause with the canonical SOV word order. The subordinate adjunct clause was marked with the resultative conjunctive suffix–key “so that” (Sohn, 1999; see Table 1 for a sample item and Figure 1 for its tree structure). The sentences are interpreted as meaning that the event denoted in the matrix clause is carried out in a manner that allows the event denoted in the subordinate clause to be performed. For example, the sentences in Table 1 are interpreted to mean that “The chair/Jinswu closed the front door so that the president/Inho could start a meeting on time.”

**Table 1:** **Example experiment item**.

**Main**	**Embedded Subj**	**W1/R1**	**W2/R2**	**W3/R3**	**W4/R4**
H	H	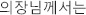	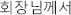	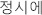	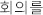
		chair-HON.TOP	president-HON.NOM	on.time-at	meeting-ACC
NH	H	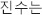	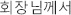	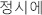	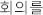
		Jinswu-TOP	president-HON.NOM	on.time-at	meeting-ACC
H	NH	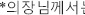	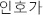	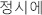	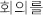
		chair-HON.TOP	Inho-NOM	on.time-at	meeting-ACC
NH	NH	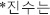	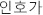	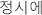	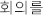
		Jinswu-TOP	Inho-NOM	on.time-at	meeting-ACC
self-paced RT		W5	W6	W7	W8
eye-tracking		R5	R6	R7	
H	H	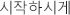	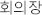	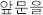	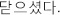
		start-HON-COMP	conference.room	front.door-ACC	closed
NH	H	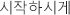	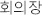	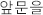	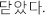
		start-HON-COMP	conference.room	front.door-ACC	closed
H	NH	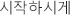	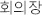	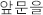	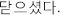
		start-HON-COMP	conference.room	front.door-ACC	closed
NH	NH	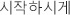	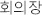	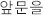	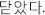
		start-HON-COMP	conference.room	front.door-ACC	closed

“*The chair/Jinswu closed the front door of the conference room so that the president/Inho could start the meeting on time.”*

The critical word position is the embedded verb (W5), which was always marked with an honorific marker –*si–*. The main clause subject (W1) and the embedded clause subject (W2) varied in their honorific features (H: Honorifiable vs. NH: Not-Honorifiable), yielding two congruous (H-H & NH-H) and incongruous conditions (H-NH & NH-NH). In Korean, personal names (e.g., Jinswu, Inho) are not honorifiable, while people of high social status (e.g., teacher, editor) are. Thus, the H conditions involved description nouns with high social status while the NH conditions involved personal names. We did not employ non-honorific description nouns (e.g., kid) for the NH conditions as two nominative marked description NPs occurring in a row (e.g., teacher-nom kid-nom…) could be interpreted as an instance of the multiple nominative construction in Korean, where the second NP can inherit the honorific features of the first NP (e.g., For the teacher_*i*_, his_*i*_ kid…) (Sohn, 1999). In addition, the H conditions used the honorific case marker –*kkeyse*, while the NH conditions used standard case markers, -*i/ka*. The main verb (W8) always occurred in the sentence final position and agreed with the main subject.

There were forty sets of experimental sentences. To make sure that the perceived incongruity is only due to the subject-verb mismatches in the honorific feature, we first conducted a norming study to control for the plausibility of the events denoted within the embedded clause with the H and the NH subjects. 20 native Korean speakers participated in the norming study. At the time of the experiment, they were enrolled as undergraduate students at Konkuk University, Korea and received ₩3000 for their participation. The norming items were created based on the embedded clause of the experimental sentences, but the verbs were modified to agree with the subject in their honorific features. For example, from the sentences with the embedded H and NH subject from Table 1, sentences like (4) and (5) were created. The norming sentences were split into two lists using a Latin-square design. Participants rated the plausibility of each sentence on a scale of 1 (sounds unlikely) to 5 (sounds plausible). A *t*-test showed that the plausibility of the two conditions was not significantly different from each other [*t*_(19)_ = 0.139, *p* < 0.89] with the mean ratings of 4.72 (*se*:0.037) and 4.724 (*se*:0.036) for the H subject and the NH subject conditions respectively.

**Table:** 

(3)	Honorifiable subject.			
	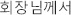	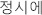	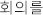	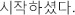
	president-HON.NOM	on.time-at	meeting-ACC	start-hon-past-decl
	“The president started the meeting on time.”			
(4)	Not-honorifiable subject.			
	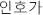	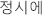	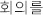	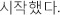
	Inho- NOM	on.time-at	meeting-ACC	start-past-decl
	“Inho started the meeting on time.”			

Our predictions are as follows. As the Korean honorific marker should agree with the verb's subject, incongruous sentences with the NH embedded subject (H-NH & NH-NH conditions) will incur processing difficulty at the embedded verb marked with –*si*– due to the mismatched honorific features compared to their congruous counterpart sentences with the H embedded subject (H-H & NH-H conditions). However, if the processing of subject-verb honorific agreement in the embedded clause is susceptible to facilitatory intrusion effects from the structurally illicit but feature-matching main subject, the H-NH condition will show less processing difficulty than the NH-NH condition, and possibly no processing difficulty, when compared to the congruous conditions (H-H & NH-H). Thus, we predict an interaction between the main and the embedded subject's honorific features, at the embedded verb position. In addition, if attraction effect is an error-driven process, the effect of a structurally illicit (main) subject will be found only for ungrammatical sentences and the onset of the effect will follow the grammaticality effect. On the other hand, if attraction effect results from general working memory principles, the effect of an illicit subject will affect the relatively initial stages of dependency formation, and will be found both for grammatical and ungrammatical sentences.

### Procedures

Four lists were created using a Latin square design. In addition to the 40 experimental sentences, there were 160 filler sentences of similar length and complexity. The experiment was run individually for each participant, using a laptop running *Linger* (Doug Rohde, MIT) in a quiet room. Stimulus presentation was word-by-word, self-paced, and non-cumulative. Participants answered a yes/no comprehension question for half of the sentences. Comprehension questions asked about the content of the target sentences. For example, for the sentences in Table 1, “Will there be a meeting on time?” was asked. For other sentences, comprehension questions asked about the content of the main clause. To illustrate, a question such as “Was the door closed?” was asked. The experiment took about 30 min.

### Data analysis

Comprehension reading times and accuracy and mean reading times for each condition are given in Tables 2, 3 respectively. Comprehension question reading times were defined as the time interval from the onset of question presentation on the screen until the response button press. Reading times were first trimmed by removing individual data points that fell above 3 standard deviations from the overall mean for a given word position, and were log-transformed. The reading time data were then analyzed using Linear Mixed Effect Regression (LMER) analysis (Baayen, 2008; Baayen et al., 2008; Jaeger, 2008). The lme4 R package (Bates et al., 2015; version 1.1-8) was used. The regression included two fixed-effect factors (the honorific features of the main and the embedded subject: H vs. NH) as well as their interaction. The fixed-effect factors were coded numerically using sum coding. For the reading time data, an LMER model was constructed for each region of interest. The comprehension accuracy rates were analyzed using a generalized LME model with a binomial distribution. The regression models incorporated crossed random intercepts for participants and items. When constructing models, we started with the maximal random effect structure, following Barr et al. (2013). When models with maximal random effect structure did not converge, we progressively simplified the random effect structure until the model converged. In Table 4, we reported in the “slope” column whether the random slope parameter corresponding to a fixed-effect factor was included in the model for participants or items. The analyses yielded coefficients, standard errors and *t*-values (*z*-values for the logit model) for each fixed effect and interaction. For the linear models, a given coefficient was judged to be significant at = 0.05 if the absolute value of *t* exceeded 2 (Baayen, 2008). For the binomial logit model, *p*-values were taken from the Z score. Finally, planned (paired) contrasts are reported using the Tukey test (using the glht function) in multcomp package (Hothorn et al., 2008; version 1.4-1) in R (R Core Team, 2015).

**Table 2:** **Mean reading times of comprehension questions and accuracy rates in Experiment 1**.

**Con1**	**Con2**	**Mean question reading times (*se*) in ms**	**Accuracy**
H	H	1593.9 (50)	94.6% (0.012)
NH	H	1386.2 (29)	97.3% (0.008)
H	NH	1502.4 (38)	96.2% (0.001)
NH	NH	1623.8 (47)	93.0% (0.013)

**Table 3:** **Mean reading times (standard errors) in Experiment 1**.

**(ms)**	**W1**	**W2**	**W3**	**W4**	**W5**	**W6**	**W7**	**W8**
H & H	526 (20)	519 (13)	560 (16)	466 (9)	444 (9)	415 (7)	401 (6)	502 (14)
NH & H	484 (16)	524 (15)	543 (14)	464 (9)	463 (11)	434 (9)	415 (7)	489 (13)
H & NH	529 (24)	484 (10)	492 (11)	434 (8)	435 (10)	453 (9)	427 (8)	511 (14)
NH & NH	476 (14)	474 (10)	521 (14)	452 (8)	522 (18)	461 (9)	431 (7)	530 (16)

*Mean Reading time results*.

Table 4**Generalized Linear Mixed Effects results for reading times in Experiment 1**.

**Table:** 

	**Estimate**	**SE**	***t***	**Slope**
**WORD 1**				
(Intercept)	6.09	0.04	149.8	
Main subj	−0.025	0.012	−2.07^*^	(p,i)
Emb subj	−0.003	0.01	−0.34	(p,i)
M^*^Emb subj	−0.0002	0.01	−0.02	(p,i)
**WORD 2**				
(Intercept)	6.13	0.04	151.7	
Main subj	−0.011	0.012	−0.92	(p,i)
Emb subj	−0.025	0.011	−2.36^*^	(p,i)
M^*^Emb subj	−0.005	0.01	−0.45	(p,i)
**WORD 3**				
(Intercept)	6.17	0.04	149.5	
Main subj	0.002	0.012	0.19	(p,i)
Emb subj	−0.035	0.011	−3.25^*^	(p,i)
M^*^Emb subj	0.013	0.011	1.19	(p,i)
**WORD 4**				
(Intercept)	6.06	0.03	181.0	
Main subj	0.012	0.008	1.57	(p,i)
Emb subj	−0.02	0.009	−2.3^*^	(p,i)
M^*^Emb subj	0.013	0.008	1.66	(p,i)
**WORD 5**				
(Intercept)	6.05	0.04	162.4	
Main subj	0.035	0.01	3.4^*^	(p,i)
Emb subj	0.008	0.009	0.89	(p)
M^*^Emb subj	0.019	0.009	2.13^*^	(p)
**WORD 6**				
(Intercept)	6.03	0.03	188.3	
Main subj	0.011	0.009	1.32	(p,i)
Emb subj	0.032	0.009	3.66^*^	(p,i)
M^*^Emb subj	−0.003	0.009	−0.35	(p,i)
**WORD 7**				
(Intercept)	5.99	0.03	215.7	
Main subj	0.014	0.007	1.9+	(p,i)
Emb subj	0.021	0.009	2.35^*^	(p,i)
M^*^Emb subj	−0.004	0.007	−0.61	(p,i)
**WORD 8**				
(Intercept)	6.13	0.05	127.8	
Main subj	0.003	0.01	0.29	(p,i)
Emb subj	0.017	0.01	1.73	(p,i)
M^*^Emb subj	0.007	0.009	0.85	−
**QUESTION**				
(Intercept)	7.23	0.05	141.4	
Main subj	−0.007	0.016	−0.4	(p,i)
Emb subj	0.018	0.015	1.22	(p,i)
M^*^Emb subj	0.037	0.014	2.63^*^	(p,i)

**Table:** 

**Accuracy**	**Estimate**	***SE***	***z***	***p***	**Slope**
(Intercept)	4.55	0.77	5.94		
Main subj	−0.156	0.264	−0.59		(p)
Emb subj	0.19	0.199	0.956		−
M^*^Emb subj	−0.416	0.201	−2.07	^*^	−

*Coefficients, standard errors, t or z-values, and p-values are reported for the main effects of the main and the embedded subject manipulation, as well as for the interaction of these two factors. The “Slope” column indicates whether the random slope parameter corresponding to the effect was included in the model for participants (p) or items (i). An asterisk indicates that the effect is significant at p < 0.05 (using the |t| > 2 criterion)*.

### Results and discussion

**Figure 2:**
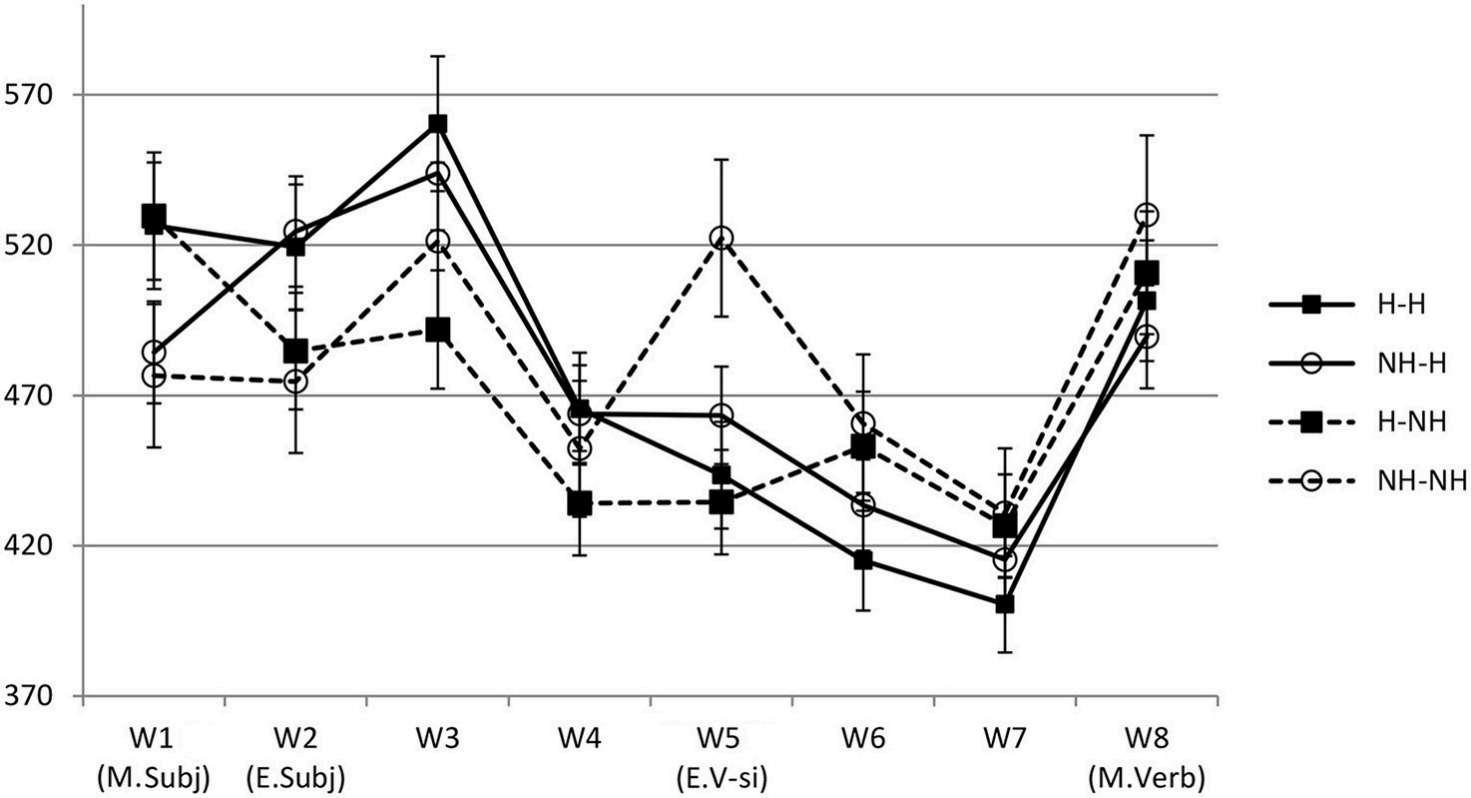
**Reading time results of Experiment 1 (self-paced reading time experiment)**.

Statistical analysis results for reading times and comprehension accuracy are given in Table 4. Figure 2 shows reading times at all word positions.

The results showed that at W1 (chair/Jinswu-top), there was a main effect of the main subject. The H-main condition (528 ms) elicited longer reading times than the NH-main conditions (480 ms), and this was probably due to longer word length in the H-main condition (6 syllables on average) than the NH-main condition (3.7 syllables on average). The effect of the main subject was no longer significant in a follow-up statistical analysis run on the residual reading times, adjusting for length (Ferreira and Clifton, 1986; β = −0.00016, *se* = 0.0098, *t* = −0.016).

At W2 (president/Inho-nom), W3 (on.time), and W4 (meeting-acc), there was a main effect of the embedded subject with the H-emb condition (W2: 522 ms; W3: 552 ms; W4: 464 ms) taking longer to read than the NH-emb condition (W2: 479 ms; W3: 506 ms; W4: 443 ms). However, as the H-emb conditions (5.2 syllables) have longer words than the NH-emb conditions (3.1 syllables) on average at W2, the main effect of the embedded subject at W2 could be due to the difference in the word length. When a follow-up statistical analysis was run on the residual reading times at W2 calculated based on the syllable lengths at the position (Ferreira and Clifton, 1986), the main effect of the embedded subject was no longer significant (β = −0.0001, *se* = 0.0091, *t* = 0.001). In addition, given the similar reading time patterns at W3 and W4, the continuing effects of the embedded subject at these word positions are most likely to be a spill-over effect from W2.

At W5 (the critical embedded verb position; start-hon-comp), there was a main effect of the main subject with the NH-main conditions (493 ms) eliciting longer reading times than its H-main counterpart (439 ms). This suggests that although honorific agreement should be local in Korean, the honorific feature mismatch between main clause subject and the embedded verb led to overall processing difficulty. Importantly, however, at W5 there was also a significant interaction of the main and the embedded subject. The reading time patterns at W5 were such that an honorific feature mismatch effect was found only in the NH-NH condition, while no such effect was found in any other conditions, including the H-NH condition (i.e., attraction condition). Indeed, *post-hoc* Tukey pairwise comparisons showed that the NH-NH condition significantly differed from both the H-H condition (*p* < 0.014) and the H-NH condition (*p* < 0.001). However, there was no significant difference among other conditions (*n.s*.). Overall, these results showed that the processing difficulty due to mismatching features in the subject-verb honorific agreement in the embedded clause was reduced or removed when there was a structurally illicit but feature-matching main subject. Thus, the results at W5 suggest that retrieval processes underlying the processing of the subject-verb honorific agreement in Korean are prone to attraction.

At W6 (the spill-over region) and W7 (sentence final main verb), there was a main effect of the embedded subject. In these word positions, the NH-emb condition (W6: 457 ms; W7: 429 ms) took longer to read than the H-emb condition (W6: 424 ms; W7: 408 ms), which could be due to mismatching honorific features between the embedded subject and its verb.

Finally, an interaction of the main and the embedded subject was significant for comprehension question response times, and for the comprehension accuracy rates. The reading times of comprehension questions and accuracy rates showed a typical similarity-based interference effect. That is, when the main and the embedded subjects were similar (H-H & NH-NH conditions), comprehension questions took longer to read and were answered with lower accuracy than when the main and the embedded subjects were different (H-NH & NH-H conditions). However, the nature of the similarity effect is not clear given the current design. While it is plausible that the similarity effect is due to the similarity in honorific features, it could be also due to the similarity in the type of a noun. To avoid the multiple nominative construction reading, we used a description noun for the H condition and a personal name for the NH condition. Previous studies have showed that the use of the same type of noun can lead to processing difficulty similar to what was found in this study (Lewis, 1996; Gordon et al., 2001, 2004, 2006). Accordingly, the apparent similarity effect could be also due to the nature of a noun used.

Overall, the results suggest that the retrieval processes underlying the processing of subject-verb honorific agreement in Korean are susceptible to facilitatory intrusion effects from the structurally illicit but feature-matching subject, with a pattern that is similar to the subject-verb number or person agreement in English. In addition, the main effect of the main clause subject at W5 suggests a possibility that the attraction effect is not limited to the ungrammatical sentences but can be found in grammatical sentences as well. However, the main effect of the main clause subject was accompanied by the interaction effect with the embedded subject, and the attraction effect was mainly observed in the ungrammatical sentences. On the other hand, grammaticality effect (i.e., the main effect of the embedded subject) was slightly delayed and was only found in spillover word positions (i.e., W6 and W7). The finding that the attraction effect precedes grammaticality effect is not compatible with the attraction as-an-error-driven process hypothesis. However, given the nature of the self-paced reading time method, the exact temporal relations of grammaticality effect and the attraction effect needs further investigations. This question was addressed using an eye-tracking method in Experiment 2.

## Experiment 2

The results of Experiment 1 showed that the retrieval processes underlying the subject-verb honorific agreement in Korean is also prone to an attraction effect of a feature-matching but structurally illicit subject, supporting the generality of the retrieval processes across languages. The detailed time course of the attraction effect in relation to that of grammaticality effect, however, needs further investigation. To this aim, Experiment 2 employed an eye-tracking method to examine the processing of the subject-verb honorific agreement in Korean.

### Participants

44 native speakers of Korean (mean age 23) participated in the study. At the time of participation, they were undergraduate students enrolled in Konkuk University, Korea and had normal or corrected-to-normal vision. They received W10000 per hour for their participation.

### Materials

The same sets of experimental sentences used in Experiment 1 were used for the eye-tracking experiment. The experimental sentences were divided into seven regions as shown in Table 1, for the purpose of analysis. Region 5 is the critical embedded verb position and Region 6 is the spill over region.

### Procedure

Four lists were created using a Latin square design, and experimental sentences in each list were pseudo-randomized along with filler sentences such that no two items from the same condition would consecutively occur. Three practice sentences were presented before the main experiment. The experiment was programmed using SR Research Experiment Builder. Participants' eye movements were tracked with an EyeLink 1000 Plus tower-mounted eye-tracker while they read experimental sentences. The tracker sampled pupil locations at a rate of 1000 Hz. The eye-tracker was fully calibrated using a standard 9 point calibration routine before the experiment started, and recalibration was performed whenever deemed necessary throughout the experiment. At the start of each trial, a black square appeared on the left side of the screen, marking the position at which the first character of the upcoming sentence would be presented. When the tracker successfully detected a participant's fixations on the black square, it was automatically replaced by the experimental stimuli. Participants were asked to read the sentences at their natural speed. As in Experiment 1, participants answered a yes/no comprehension question for half of the sentences.

### Data analysis

Prior to analysis, we pooled short fixations of less than 80 ms and merged them into larger fixations within the distance of the visual angle of 0.05. If there was no fixation nearby, the short fixations were removed. Fixations longer than 1200 ms were also removed. We report below three eye-fixation measures. First pass reading times are the sum of all fixations in a given region, from the first entry into the region, before the eyes leave the region either to left or right. Go-past times or Regression path times are the sum of all fixations spent in a region from the first entry into the region from the left until the first exit of the region to the right. Total time is the sum of all fixations in the region. We excluded the trials in which the region was skipped in initial reading from the analysis of First-pass reading time or Go-past times, and excluded the trials in which the region was not fixated at all from the analysis of Total Time.

For data analysis, reading times were first log-transformed and analogous analysis procedures to Experiment 1 were applied for the reading times of each region defined in Table 1. Region 1 and Region 2 differed between the Honorific and Non-honorific conditions (e.g., *president* vs. *Inho*) and the analyses in Experiment 1 showed clear length effect such that the H-main condition elicited longer reading times than the NH-main conditions due to longer word length in the former than the latter condition. Thus, the analyses for R1 and R2 were based on residual reading times (Ferreira and Clifton, 1986). Below, we report the data analysis results.

### Results and discussion

Mean comprehension accuracy and reading times for each condition are given in Tables 5, 6 respectively, and statistical analysis results are given in Table 7.

**Table 5:** **Mean comprehension accuracy rates and statistical analysis results in Experiment 2**.

	**Mean (se)**		**Estimate**	***SE***	***z***	***p***	**Slope**
H & H	90.0% (0.014)	Intercept	2.59	0.34	7.67	0.001	
NH & H	88.6% (0.015)	Main sub	−0.26	0.4	−0.67	n.s.	(p)
H & NH	89.5% (0.015)	Emb subj	−0.03	0.34	−0.09	n.s.	
NH & NH	89.1% (0.015)	M ^*^ E subj	0.07	0.46	0.16	n.s.	

**Table 6:** **Means (and standard errors), aggregated by participants, for first pass, go-past, and total times in milliseconds in Experiment 2**.

**(ms)**	**Region1**	**Region2**	**Region3**	**Region4**	**Region5**	**Region6**	**Region7**
**FIRST PASS**							
H & H	438 (12)	350 (10)	246 (6)	264 (6)	303 (9)	410 (10)	318 (15)
NH & H	356 (12)	340 (11)	260 (6)	261 (6)	308 (8)	439 (12)	304 (12)
H & NH	473 (13)	288 (8)	267 (7)	272 (8)	311 (8)	411 (11)	323 (12)
NH & NH	371 (10)	277 (8)	265 (7)	271 (7)	328 (9)	454 (13)	307 (13)
**GO-PAST**							
H & H	438 (12)	485 (18)	362 (15)	374 (17)	356 (15)	983 (62)	1931 (124)
NH & H	356 (12)	501 (18)	361 (12)	320 (12)	358 (13)	944 (50)	1817 (129)
H & NH	473 (13)	358 (20)	343 (12)	368 (15)	372 (13)	956 (58)	1783 (119)
NH & NH	371 (10)	436 (17)	371 (16)	365 (15)	421 (19)	1189 (61)	2119 (151)
**TOTAL TIME**							
H & H	927 (30)	919 (35)	507 (17)	462 (17)	505 (20)	830 (36)	479 (22)
NH & H	685 (24)	882 (31)	492 (20)	432 (16)	500 (18)	828 (37)	425 (19)
H & NH	846 (30)	622 (22)	554 (22)	484 (18)	553 (24)	797 (33)	486 (23)
NH & NH	831 (30)	839 (31)	575 (18)	496 (20)	593 (24)	895 (34)	443 (19)

**Table 7:** **Generalized Linear Mixed Effects results for reading times in Experiment 2**.

		**First pass**				**Go-past**				**Total time**			
		**Coeff**.	***SE***	***t***	**Slope**	**Coeff**.	***SE***	***t***	**Slope**	**Coeff**.	***SE***	***t***	**Slope**
R1 “*chair/Jinswu”*	Intercept	0.0001	0.013	0.001		−	−	−	−	−0.001	0.018	−0.003	
	Main subj	0.0001	0.014	0.007	(p,i)	−	−	−	−	0.001	0.017	0.013	(p,i)
	Emb subj	0.0005	0.01	0.047	(p,i)	−	−	−	−	−0.001	0.013	−0.015	(p,i)
	M ^*^ E subj	−0.006	0.01	−0.622	(p,i)	−	−	−	−	0.069	0.011	6.275^*^	-
R2 “*president/Inho”*	Intercept	0.0003	0.016	0.018		0.001	0.019	0.011		−0.001	0.023	−0.019	
	Main subj	−0.0002	0.012	−0.018	(p,i)	−0.001	0.015	−0.088	(p,i)	−0.002	0.018	−0.101	(p,i)
	Emb subj	−0.0006	0.014	−0.044	(p,i)	−0.001	0.013	−0.034	−	0.001	0.017	0.042	(p,i)
	M ^*^ E subj	0.0011	0.013	0.091	(p,i)	0.029	0.013	2.226^*^	−	0.084	0.017	4.885^*^	(p,i)
R3 “*on time”*	Intercept	5.46	0.03	196.45		5.69	0.04	162.45		6.07	0.05	124.55	
	Main subj	−0.01	0.01	−0.75	(p,i)	−0.02	0.01	−1.11	(p,i)	−0.002	0.02	−0.1	(p,i)
	Emb subj	−0.02	0.01	−1.7	(p,i)	−0.001	0.01	−0.06	−	−0.06	0.02	−3.32^*^	(p,i)
	M ^*^ E subj	−0.02	0.01	−1.67	(p,i)	0.001	0.01	0.001	−	0.03	0.02	1.5	(p,i)
R4 “*meeting-ACC”*	Intercept	5.48	0.03	183.33		5.68	0.04	148.02		5.93	0.05	120.91	
	Main subj	0.002	0.01	0.17	(p,i)	0.02	0.01	1.63	(p,i)	0.02	0.02	1.05	(p,i)
	Emb subj	−0.01	0.01	−0.5	(p,i)	−0.03	0.02	−1.76	(p,i)	−0.03	0.02	−2.08^*^	(p,i)
	M ^*^ E subj	0.01	0.01	0.54	(p,i)	0.03	0.01	1.74	(p,i)	0.02	0.02	1.21	(p,i)
R5 “*start-HON”*	Intercept	5.6	0.04	143.7		5.73	0.04	140.2		6.05	0.06	109.84	
	Main subj	−0.02	0.01	−1.24	(p,i)	−0.02	0.02	−1.04	(p,i)	−0.03	0.02	−1.82	(p,i)
	Emb subj	−0.02	0.01	−1.51	(p,i)	−0.04	0.02	−2.36^*^	(p,i)	−0.05	0.02	−2.76^*^	(p,i)
	M ^*^ E subj	0.003	0.01	0.27	(p,i)	0.01	0.02	0.39	(p,i)	0.02	0.02	1.07	(p,i)
R6 “conference room”	Intercept	5.91	0.04	164.07		6.5	0.07	91.47		6.48	0.07	97.25	
	Main subj	−0.04	0.01	−2.53^*^	(p,i)	−0.07	0.02	−2.86^*^	(p,i)	−0.03	0.02	−1.8	(p,i)
	Emb subj	−0.003	0.02	−0.2	(p,i)	−0.06	0.02	−2.47^*^	(p,i)	−0.03	0.02	−1.57	(p,i)
	M ^*^ E subj	0.01	0.01	0.65	(p,i)	0.04	0.02	2.08^*^	(p,i)	0.04	0.01	2.81^*^	(p,i)
R7 “door-ACC closed”	Intercept	5.5	0.05	104.66		7.06	0.1	71.84		5.8	0.07	87.67	
	Main sub	0.03	0.01	1.98+	(p,i)	−0.01	0.02	−0.61	(p,i)	0.05	0.02	2.73^*^	(p,i)
	Emb subj	−0.01	0.01	−0.89	(p,i)	−0.01	0.02	−0.23	(p,i)	−0.02	0.02	−0.97	(p,i)
	M ^*^ E subj	−0.02	0.01	−1.34	−	0.05	0.02	2.24^*^	(p,i)	−0.001	0.02	−0.09	(p,i)

*Coefficients, standard errors, t or z-values, and p-values are reported for the main effects of the main and the embedded subject manipulation, as well as for the interaction of the main and the embedded subject manipulation, as well as for the interaction of these two factors. The “Slope” column indicates whether the random slope parameter corresponding to the effect was included in the model for participants (p) or items (i). An asterisk indicates that the effect is significant at α = 0.05 (using the |t| > 2 criterion)*.

Region 1 (chair/Jinswu-top) and Region 2 (president/Inho-nom).

There was a significant interaction between the main subject and embedded subject in Total times at R1, and in Go-past times and Total times at R2. These reading times showed a typical similarity-based interference effect, such that the sentences took longer to read when the main and the embedded subject were similar (H-H & NH-NH conditions) than when they were not (H-NH vs. NH-H). *Post-hoc* pairwise comparisons of the Total times showed significant differences between these two types (all *ps* < 0.01; see Table 6). Pairwise comparisons of Go-past times in R2, on the other hand, did not yield any significant differences although the reading times showed numerically similar patterns as Total times.

Region 3 (on.time) and Region 4 (meeting-acc).

At R3 and R4, there was a significant main effect of the embedded subject in total times. In these word positions, the NH condition took longer to read than the H condition (R3: 564 vs. 499 ms; R4: 490 vs. 447 ms; R5: 573 vs. 502 ms).

Region 5 (the critical embedded verb position).

**Figure 3:**
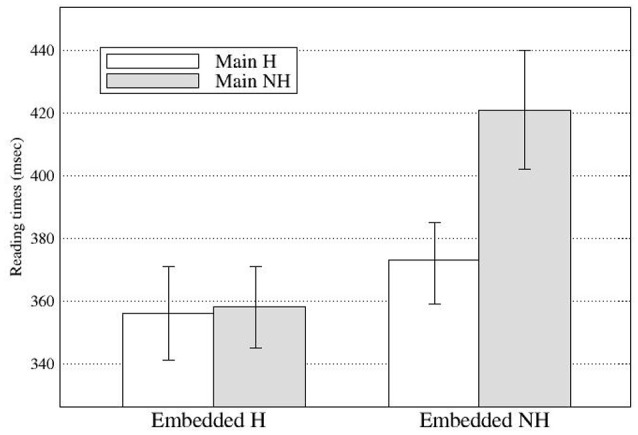
**Regression Path duration at Region 5 in Experiment 2 (Error bars show standard errors)**.

At R5, there was a main effect of the embedded subject in Go-past times and Total times (see Figure 3), with the NH-emb conditions taking longer to read than the H-emb conditions (Go-past times: 396 vs. 357 ms; Total times: 573 vs. 502 ms). The effect in Go-past times suggests that the grammaticality effect was not delayed in the eye-tracking experiment, unlike in Experiment 1.

Region 6 (the spill over region).

**Figure 4:**
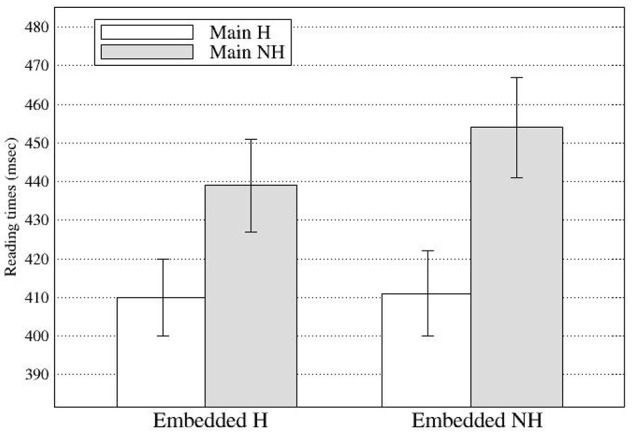
**First pass reading times at Region 6 in Experiment 2 (Error bars show standard errors)**.

**Figure 5:**
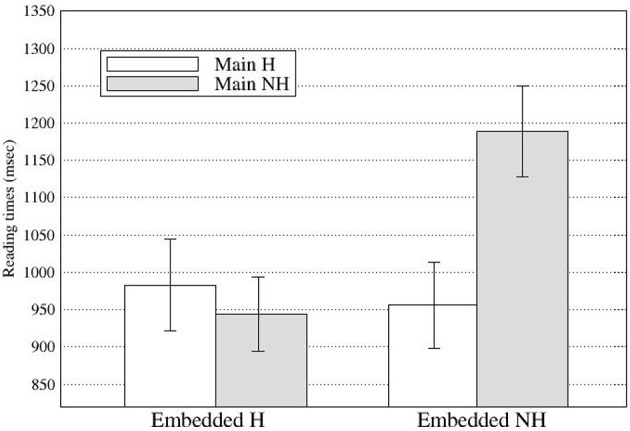
**Regression path times at Region 6 in Experiment 2 (Error bars show standard errors)**.

At R6, there was a significant main effect of the main clause subject in First pass and Go-past times with longer reading times for the NH main subject condition (First pass: 446 ms; Go-past: 1066 ms) than for the H main subject condition (First pass: 410 ms; Go-past: 969 ms; see Figure 4). Although slightly delayed compared with Experiment 1, these effects confirm the observation in Experiment 1 that the processing of the subject-verb honorific agreement in an embedded clause was affected by the main clause subject. In addition, the results clearly suggest that the effect of the attractor was not limited to the ungrammatical conditions but can be found in the grammatical conditions as well. At R6, there was also a main effect of the embedded subject in Go-past times with the NH-emb condition taking longer to read than the H-emb condition (1072 vs. 963 ms), due to spill-over effect of grammaticality effect found at R5. Finally, a significant interaction between the main subject and embedded subject was also found in Go-past times and Total times. The reading time patterns were such that the honorific feature mismatch costs in the embedded subject-verb agreement dependency were modulated by the honorific features of the main subject. Pair-wise comparisons of Go-past times showed that the mismatch cost was only evident in the NH-NH condition, which took significantly longer to read than the H-H condition (*p* < 0.004), the NH-H condition (*p* < 0.02) and the H-NH condition (*p* < 0.001), and that these three conditions did not differ from each other (see Figure 5). *Post-hoc* analyses of Total times showed also similar results, with the NH-NH condition taking significantly longer to read than the NH-H condition (*p* < 0.03) and the H-NH condition (*p* < 0.003), while the other three conditions—the H-H condition, the NH-H condition (congruous conditions), and the H-NH condition (the attraction condition)—did not differ from each other.

Region 7 (sentence final region).

At R7, there was a main effect of the main clause subject in First pass times (albeit marginal) and Total times, with longer reading times for the H main subject condition (First pass: 320 ms; Total times: 482 ms) than for the NH main condition (First pass: 305 ms; Total times: 433 ms). These effects suggest that the honorific features of the main clause subject affect the processing of the embedded verb but the direction of the effect is opposite to that found at R6. We believe that the effect might reflect processing difficulty during later parsing stages. In particular, the effect could be related to the processing difficulty associated with the parser's correct rejection of the main clause subject as a licit subject for the embedded verb. Accordingly, sentences could have taken longer to read when they involved a main clause subject which matched an embedded verb in features, as these sentences could have required more effort to reject an incorrect interpretation. In addition, there was a significant interaction between the main subject and embedded subject in Go-past times at R7. The reading times showed numerically similar patterns to those found in Go-past times at R6, such that the honorific feature mismatch costs in the embedded subject-verb agreement dependency were modulated by the honorific features of the main subject. However, unlike at R6, no significant difference was found in the pairwise comparisons at R7.

Overall, Experiment 2 replicated the main findings of Experiment 1; the retrieval processes underlying the subject-verb honorific agreement were prone to attraction from a structurally illicit argument. In addition, as in Experiment 1, a main effect of the main clause subject was found, but the results of Experiment 2 more clearly suggest that the attraction effect is not limited to ungrammatical sentences but can be found in grammatical sentences as well. The results of Experiment 2 further showed that the main effect of the embedded subject (i.e., grammaticality effect) was observed earlier than the effect of the main clause subject (i.e., attraction effect in the form of either the main effect of the main clause subject or the interaction of the main and the embedded subject).

## General discussion and conclusion

We initially predicted that if the retrieval processes underlying the subject-verb honorific agreement in Korean are similar to those involved during the processing of the subject-verb person or number agreement in English, attraction effects would be also observed in Korean as well. In addition, we predicted that if attraction effect is an error-driven process (Wagers et al., 2009), attraction effect would be found only for ungrammatical sentences and the onset of the effect would follow the grammaticality effect. The predictions were partly confirmed. First, there was clear evidence that the honorific features of main clause subject affected the processing of the embedded verb. However, the effect of a structurally illicit (main) subject was not limited to the ungrammatical sentences but was across-the-board. For example, the main effect of the main clause subject in First-pass reading times at the spillover region (R6) in Experiment 2 clearly showed that honorific feature matches between the main clause subject and the embedded verb led to overall processing facilitation and mismatches to overall processing difficulty, regardless of honorific features of the licit (embedded) subject. On the other hand, no clear evidence was found for the temporal relations between the attraction and grammaticality effect. Below we discuss these findings and their implications in detail.

Although the honorific system in Korean involves a kind of subject-verb agreement similarly to subject-verb number or person agreement in English, the honorific dependency in Korean is not obligatory but is based on pragmatic features, unlike grammaticalized subject-verb agreement in English (see Introduction for details). Nonetheless, intrusion effects found in Experiments 1, 2 were strikingly similar to the patterns found with subject-verb agreement in English. These results support the hypothesis that the representations formed during the processing of honorific agreement in Korean are content-addressable. Applied to the current study, several cues, such as honorific features, grammatical roles, and structural information could have been used, based on the features of the embedded verbs. This could have allowed direct access to a potential target in memory, where honorific features were signaled by pragmatic or world knowledge (e.g., grandpa vs. kid), grammatical role by a case marker (e.g., nominative marking), and structural information by positional information (e.g., the 1st NP as the main subject and the 2nd NP as the embedded subject). Accordingly, the structurally illicit (main) subject with partial feature matches could have been activated in Experiments 1, 2, leading to facilitatory intrusion effects, with a pattern that is similar to subject-verb agreement in English.

Concerning the nature of attraction effects, the results are more compatible with the hypothesis that attraction effects result from general working memory principles. As discussed in Introduction, the attraction effect has been proposed to be an error-driven processing mechanism, based on two observations, namely that (i) the onset of the attraction effect has been observed to follow the grammaticality effect, and (ii) that the attraction effect has been observed only for ungrammatical sentences (Pearlmutter et al., 1999; Wagers et al., 2009; Dillon et al., 2013; Lago et al., 2015; cf. Van Dyke, 2007). The results from the current study, however, do not provide clear and consistent evidence for the temporal relations between the attraction and grammaticality effect. In Experiment 1, the grammaticality effect was first found at the spillover region, one word after the critical embedded verb position while the attraction effect was first found at the critical verb position. However, in Experiment 2, the grammaticality effect was first found in the Go-past times at the critical region while the attraction effect was first found in the First-pass times at the spillover region (in the form of a main effect of the main clause subject) and was also found in the Go-past times at the same region (in the form of the interaction of the main and the embedded subject). Thus, the results of Experiments 1, 2 provided a slightly different picture on the time course of the attraction effect in relation to that of grammaticality effect. One thing to note, however, is that even though there was a main effect of the embedded subject at the critical region (R5) in Experiment 2 (i.e., a grammaticality effect), the relative processing difficulty of NH-emb compared to H-emb was mainly observed when the main subject was also non-honorific, despite the lack of an interaction in this region. When the main subject was honorific, however, the processing difficulty of NH-emb sentences was greatly reduced (see Figure 4), a typical attraction effect. If so, the results from Experiments 1, 2 do not seem conflicting with each other. However, although no clear evidence was found for the temporal relations between the attraction and grammaticality effect in either of the experiments, the results from the current study clearly showed that the attraction effect was found for grammatical sentences as well as for ungrammatical sentences. As grammatical sentences do not contain errors, the attraction effect in grammatical sentences is not compatible with the attraction-as-an-error-driven processing hypothesis. Instead, the results are more compatible with the hypothesis that the attraction effect results from general working memory principles. That is, during dependency resolution, any potential target item in memory would be activated if it has a feature that matches the retrieval cue. If its activation level exceeds the retrieval threshold, an attraction effect could result.

In summary, the current study investigated the processing of subject-verb honorific agreement in Korean. The results showed that the attraction effect was found for grammatical as well as ungrammatical sentences. The overall results support the content-addressable-direct retrieval model. In addition, the clear attraction effect in the grammatical sentences suggest that the attraction effect does not solely arise as the result of an error-driven process, but is likely also to result from general mechanisms of retrieval processes of activating of potential items in memory.

## Author contributions

NK and PS were both involved in designing the experiments and writing up the paper. NK carried out the experiments and analyzed the data.

### Conflict of interest statement

The authors declare that the research was conducted in the absence of any commercial or financial relationships that could be construed as a potential conflict of interest. The reviewer SM and handling Editor declared their shared affiliation, and the handling Editor states that the process nevertheless met the standards of a fair and objective review.

## Footnotes

^1^This is among the properties that have led to Korean being called a “discourse oriented language”, which may potentially lead to a lower weighting of structural cues, relative to other languages (see Kwon and Sturt, 2013; though see also Kwon et al., 2013, for evidence that structural constraints are used on-line in Korean).

^2^The following abbreviations are used to label the linguistic terms. top: Topic, nom: Nominative, hon: Honorific, acc: Accusative, comp: Complementizer.
